# RETRACTED: Li et al. Uric Acid Causes Pancreatic β Cell Death and Dysfunction via Modulating CHOP-Mediated Endoplasmic Reticulum Stress Pathways. *Diseases* 2025, *13*, 213

**DOI:** 10.3390/diseases14110406

**Published:** 2026-11-06

**Authors:** Xueyan Li, Yunan Chen, Lei Su, Jialin He

**Affiliations:** 1 The Key Laboratory of Environmental Pollution Monitoring and Disease Control, Department of Nutrition, School of Public Health of Guizhou Medical University, Guiyang 561000, China; 2 Guangdong Provincial Key Laboratory of Food, Nutrition and Health, Department of Nutrition, School of Public Health, Sun Yat-sen University (Northern Campus), Guangzhou 510000, China

The Journal retracts the article “Uric Acid Causes Pancreatic β Cell Death and Dysfunction via Modulating CHOP-Mediated Endoplasmic Reticulum Stress Pathways” [1] cited above.

Following publication, the authors raised concerns with the Editorial Office regarding the authorization and provenance of the data used in this publication [1].

In accordance with the journal’s standard procedures, the Editorial Office and Editorial Board conducted an investigation. Based on the documentation available, the investigation could not establish the provenance of the study data or confirm the accuracy of the authorship information. In addition, indications of inappropriate image editing were identified in the supporting materials provided during submission. The concerns could not be fully resolved because the complete original data required to verify the findings in this publication [1] were not available for evaluation.

Consequently, the Editorial Board decided to retract this publication in accordance with MDPI’s retraction policy.

This retraction was approved by the Editor-in-Chief of the journal *Diseases*.

The authors have been informed of this retraction and have indicated their agreement with this decision.
